# Altered cell function and increased replication of rhinoviruses and EV-D68 in airway epithelia of asthma patients

**DOI:** 10.3389/fmicb.2023.1106945

**Published:** 2023-03-01

**Authors:** Manel Essaidi-Laziosi, Léna Royston, Bernadett Boda, Francisco Javier Pérez-Rodriguez, Isabelle Piuz, Nicolas Hulo, Laurent Kaiser, Sophie Clément, Song Huang, Samuel Constant, Caroline Tapparel

**Affiliations:** ^1^Department of Microbiology and Molecular Medicine, Faculty of Medicine, University of Geneva, Geneva, Switzerland; ^2^Epithelix Sàrl, Plan les Ouates, Geneva, Switzerland; ^3^Division of Infectious Diseases, Geneva University Hospital, Geneva, Switzerland; ^4^Service for Biomathematical and Biostatistical Analyses, Institute of Genetics and Genomics, University of Geneva, Geneva, Switzerland

**Keywords:** rhinovirus, enterovirus-D68, asthma, airway epithelial barrier, viral replication

## Abstract

**Introduction:**

Rhinovirus (RV) infections constitute one of the main triggers of asthma exacerbations and an important burden in pediatric yard. However, the mechanisms underlying this association remain poorly understood.

**Methods:**

In the present study, we compared infections of *in vitro* reconstituted airway epithelia originating from asthmatic versus healthy donors with representative strains of RV-A major group and minor groups, RV-C, RV-B, and the respiratory enterovirus EV-D68.

**Results:**

We found that viral replication was higher in tissues derived from asthmatic donors for all tested viruses. Viral receptor expression was comparable in non-infected tissues from both groups. After infection, ICAM1 and LDLR were upregulated, while CDHR3 was downregulated. Overall, these variations were related to viral replication levels. The presence of the CDHR3 asthma susceptibility allele (rs6967330) was not associated with increased RV-C replication. Regarding the tissue response, a significantly higher interferon (IFN) induction was demonstrated in infected tissues derived from asthmatic donors, which excludes a defect in IFN-response. Unbiased transcriptomic comparison of asthmatic versus control tissues revealed significant modifications, such as alterations of cilia structure and motility, in both infected and non-infected tissues. These observations were supported by a reduced mucociliary clearance and increased mucus secretion in non-infected tissues from asthmatic donors.

**Discussion:**

Altogether, we demonstrated an increased permissiveness and susceptibility to RV and respiratory EV infections in HAE derived from asthmatic patients, which was associated with a global alteration in epithelial cell functions. These results unveil the mechanisms underlying the pathogenesis of asthma exacerbation and suggest interesting therapeutic targets.

## 1. Introduction

Rhinoviruses (RVs) are among the most frequent pathogens in human worldwide, involved in more than 50% of common colds. Members of the *Enterovirus* genus, those small non-enveloped positive-stranded RNA viruses are classified into three species: RV-A, RV-B, and RV-C and can be further divided according to their receptor usage. RVs from the major group (most RV-As and all RV-Bs) bind ICAM1 (Greve et al., 1989), RV-As from the minor group bind the low-density lipoprotein receptor (LDLR; Hofer et al., 1994) and RV-Cs use cadherin related family member 3 receptor (CDHR3), expressed on ciliated airway cells (Bochkov et al., 2015; Everman et al., 2019). Some other non-RV enteroviruses (EVs), including EV-D68, share biological properties with RVs such as acid lability and optimal growth at 33°C and induce respiratory symptoms similar to RVs (Royston and Tapparel, 2016).

It is widely accepted that respiratory and particularly RV infections constitute a major trigger of asthma exacerbations and a risk factor for asthma development (Gern, 2010; Jackson and Gern, 2022). Concerning EV-D68, an association between EV-D68-related symptoms severity and a history of asthma has also been reported (Moss, 2016; Hayashi et al., 2018; Korematsu et al., 2018). The mechanisms linking viral infections to asthma exacerbation remain, however, poorly understood. While upper respiratory tract RV infections are not increased in asthmatic patients, more frequent and more severe lower respiratory tract infections are observed (Corne et al., 2002). How viral infections contribute to these clinical manifestations remains unknown. A dysregulated immune and particularly IFN-response upon infection has been shown in asthmatic patients (Contoli et al., 2006; Edwards et al., 2013; Zhu et al., 2019; Jackson and Gern, 2022; Liew et al., 2022), but remains controversial (Baraldo et al., 2012; Sykes et al., 2014; Da Silva et al., 2017; Hansel et al., 2017; Sopel et al., 2017; Jazaeri et al., 2021; Yang et al., 2021).

RV-As and RV-Cs are more frequently detected in childhood asthma exacerbations than RV-Bs. This could be explained by the difference of virulence between isolates of distinct species, but remains hypothetical (Iwane et al., 2011; Lee et al., 2012). RV-A, B, and C may induce distinct host antiviral responses, possibly through the use of different cellular receptors. A single-nucleotide polymorphism (SNP) in *CDHR3*, the RV-C receptor, is associated with greater risk of asthma hospitalizations in homozygous and heterozygous children (Bonnelykke et al., 2014; Kanazawa et al., 2017; Bonnelykke et al., 2018). This SNP (rs6967330) results in a C529Y amino acid (aa) change in the CDHR3 protein, associated with increased expression at the cell surface upon transfection, favoring RV-Cs infection (Bochkov et al., 2015). Increased expression of the mutated allele was also reported in human bronchial epithelial cells cultured at the air-liquid interface (ALI) (Basnet et al., 2019). Accordingly, the *CDHR3* risk allele was associated with increased RV-C infection incidence in two birth cohorts (Bonnelykke et al., 2018). However, the interaction between RV-C and this receptor and the role of this interaction in asthma exacerbations remains speculative. Recent studies have reported different consequences of *CDHR3* mutation on tissue differentiation, protein subcellular localization, and RV-C binding (Basnet et al., 2019; Everman et al., 2019). Everman and colleagues found that *CDHR3* knockdown affected RV-C binding but not replication and suggested that RV-Cs use a coreceptor for infection (Everman et al., 2019; Lutter and Ravanetti, 2019). Regarding major group RVs, ICAM1 expression is very low in the airways but increases upon inflammation (Bianco et al., 2000). Similarly, LDLR expression may vary in response to inflammation (Zhang et al., 2016). The asthma-associated inflammatory response could enhance the accessibility of viral receptors and improve infectivity (Bochkov and Gern, 2016). However, RV-Bs that are less frequently associated with asthma exacerbations, also use ICAM1 to infect cells. Further research is needed to better define the role of viral receptors in RV-A, RV-B, and RV-C-induced asthma exacerbations.

In this study, we aimed to explore the involvement of different RVs and of EV-D68 in asthma and assess the role of viral receptors and innate immune induction, using human airway epithelia (HAE) and clinical viral isolates. We highlighted overall increased viral replication in tissues from asthmatic patients, but could not link receptor expression or IFN-induction with this phenotype. Unbiased transcriptomic analysis showed basic morphological and physiological differences between tissues from asthmatic versus control donors, even in absence of infection, an observation supported by diminished mucociliary clearance (MCC) and increased mucus secretion in asthma-derived tissues. Our observations suggest an alteration in the mechanical defense of the respiratory mucosae in tissues derived from asthmatic patients, resulting in increased permissiveness and susceptibility to RV or respiratory EV infections.

## 2. Materials and methods

### 2.1. Human airway epithelia

HAE (“MucilAir”^1^) were reconstituted from airway cells obtained from patients undergoing surgical nasal polypectomy (for nasal tissues) or lung lobectomy (for bronchial tissues). Patients presenting no atopy, asthma, allergy, or other known respiratory comorbidity were used as controls (Supplementary Table S1). All experimental procedures were explained, and all subjects provided informed consent. The study was conducted according to the Declaration of Helsinki on biomedical research (Hong Kong amendment, 1989), and the research protocol was approved by the local ethics committee (commission cantonale d’éthique de la recherche CCER from Geneva). Cultures are performed in an ALI system according to the procedure previously detailed in (Essaidi-Laziosi et al., 2018). Once differentiated, epithelia contain ciliated, goblet, and basal cells, with a pseudostratified structure and mucociliary clearance functions.

### 2.2. Viral stocks and tissue infection

Anonymized clinical samples were screened by semi-quantitative real-time PCR (RT-sqPCR; Ambrosioni et al., 2014) and RV/EV were subtyped by sequencing as previously described (Tapparel et al., 2011; Essaidi-Laziosi et al., 2018). A respiratory EV (EV-D68), a major group RV (RV-A16), a minor group RV (RV-A49), and representatives of the B (RV-B48) and C (RV-C15) species were selected. Viral stocks were produced directly in MucilAir to avoid any adaptation in immortalized cells and titrated as described (Essaidi-Laziosi et al., 2020). Serial dilutions were performed in MucilAir to evaluate viral infectious doses within each stock. The viral inoculum was then normalized according to the determined endpoint, which corresponds to the highest inoculum dilution allowing virus replication as described (Essaidi-Laziosi et al., 2020).

Tissues were infected as previously described (Essaidi-Laziosi et al., 2018, 2020). For each virus, the inoculum was normalized based on the infectious titer to contain identical doses of infectious particles (MOI of ~0.001 per accessible cell). Four hours after inoculation, tissues were washed 3 times with PBS. At various times post-infection, 200 μL of medium was applied on the apical surface during 20 min at 33°C for sample collection. Basal medium was collected at the same time and replaced with 500 μL of fresh medium.

### 2.3. Viral load quantification, gene expression quantification, and *CDHR3* genotyping

RNA was extracted (E.Z.N.A viral RNA kit I, Omega, R687402), and quantified by one-step real-time quantitative PCR (RT-qPCR) with the QuantiTect kit (Qiagen) in a StepOne ABI thermocycler (Essaidi-Laziosi et al., 2018).

Gene expression of the receptors and IFNs at 4dpi was determined by semi-quantitative RT-PCR (RT-sqPCR) on total RNA extracted from tissue lysates using total RNA extraction kit (E.Z.N.A total RNA kit I, Omega, R8334A) and normalized to RNAseP housekeeping gene. IFNλ1 mRNA was amplified using primers and probe (Fwd GGACGCCTTGGAAGAGTCACT, rev AGAAGCCTCAGGTCC CAATTC and probe AGTTGCAGCTCTCCTGTCTTCCCCG) as previously described (Dolganiuc et al., 2012), while mRNAs from CDHR3, ICAM1, LDLR, ISG15, IFNα, IFNβ, and RNAseP were amplified using specific gene expression assay kits (Thermo Fisher Scientific, ref. 4331182, Cat N° Hs00541677_m1, Hs00164932_m1, Hs00181192_m1, Hs01921425, Hs04190680_gH, Hs01077958_s1, and 4403326). Fold changes were calculated after normalization with the RNAseP housekeeping gene with the 2(-Delta Delta C(T)) (Livak and Schmittgen, 2001). Regarding CDHR3 genotyping, the gene was amplified by PCR from extracted DNA and then sequenced using specific primers (Fwd ATTCCTCCAGCCAGAACCCG and Rev. TGTTTCTCACCACATCCGCAG).

### 2.4. ELISA and Western blot

Interferon lambda (IFNλ1/ λ3, IL-29 /IL-28B) was measured in the basal medium by ELISA (R&D DY1598B-05) according to the manufacturer’s instructions.

Western blot assays were performed as previously described (Essaidi-Laziosi et al., 2014). Infected and non-infected tissues from healthy and asthmatic donors were lysed using RIPA buffer (NaCl 150 mM, EDTA 1mM, Tris HCl pH = 7.4 50mM, NP40 1%, SDS 0.1%, and Sodium deoxycholate 1%) containing protease inhibitors (Roche, 04693159001). Cell lysates were clarified and resuspended in SDS-PAGE sample buffer and electrophoresed on 8 or 10% SDS polyacrylamide gel. Gel transfer was made onto a polyvinylidene difluoride membrane (PVDF, Biorad, 1,620,177) using Trans-Blot SD Transfer Cell (Biorad). The membranes were first blocked with 5% skim milk (AppliChem) in TTBS (20 mM Tris HCl, pH 7.5, 500 mM NaCl, and 0.05% Tween 20) at RT for 30 min and then incubated with Anti-ICAM1 (diluted 1/500, Abcam, ab2213), -LDL-R (diluted 1/1,000, R&D systems, AF2148), CDHR3 (diluted 1/500, Sigma-Aldrich, HPA011218), and Actin (diluted 1/1,000 Millipore, MAB1501) primary antibodies overnight at 4°C. The membranes were then washed 3 times with TTBS and incubated at RT for 1 h with corresponding anti-mouse or anti-rabbit horseradish peroxidase (HRP)-coupled secondary antibodies (Cell Signaling). Membranes were washed and viral receptors were detected using an enhanced chemiluminescence solution (ECL, ref. K-12043-D10 Western Bright Sirius Advansta) for 2 min. Immunoblot images were acquired using Fujifilm LAS 4000 luminescence imager.

### 2.5. Transcriptomic analysis

Infected and non-infected tissues were lysed in 800 μL of trizol (Ambion, 5,596,018) and RNA was extracted according to the manufacturer’s instruction. Total RNA was quantified with Qubit (Life Technologies) and RNA integrity was assessed with a Bioanalyzer (Agilent Technologies). The TruSeq Stranded Total RNA kit with Ribo-Zero Gold was used for the library preparation with 150ng of total RNA as input. The 18 libraries were pooled at equimolarity and loaded at 8.5 pM for clustering on a single-read Illumina Flow cell.

Library molarity and quality for all samples were assessed with a Qubit and a Tapestation using a DNA High sensitivity chip (Agilent Technologies). All 100-base sequencing was performed using the TruSeq SBS HS v3 chemistry on an Illumina HiSeq 2,500 sequencer. The sequencing raw data are available at GEO with accession number GSE222129.

Quality control of the resulting reads was done with FastQC and the reads mapped to the Homo sapiens UCSC hg38 genome with the STAR program (version 2.5.2a; Dobin et al., 2013), and count tables containing the number of mapped reads per gene were produced with featureCounts (version 1.6.0). Count tables were then imported into R (version 2.13) to do the differential expression analysis with edgeR (version 3.12.1; Robinson et al., 2010). Data were filtered for genes with weak expression level (average CPM < 1). Library sizes were adjusted with a scaling factor calculated using a trimmed mean of M-values (TMM) between each pair of samples. The common dispersion and tagwise dispersions were estimated with the estimateDisp function. After negative binomial glm fitting the quasi-likelihood (QL), F-test was applied for the testing procedure. The significantly differentially expressed gene lists (FDR < 0.05) obtained with this procedure were then used to do GO enrichment analysis on the cellular component subset of GO term database with ClusterProfiler functions (version 2.4.3) (Yu et al., 2012).

### 2.6. Mucociliary clearance, mucin measurement with enzyme-linked lectin assay

The mucociliary clearance was monitored using a Sony XCD-U100CR camera connected to an Olympus BX51 microscope with a 5× objective. Polystyrene microbeads of 30 μm diameter (Sigma, 84,135) were added on the apical surface of MucilAir. Microbeads movements were video tracked at 2 frames per second for 30 images at room temperature. Three movies were taken per insert. Average beads movement velocity (μm/s) was calculated with the ImageProPlus 6.0 software.

Mucin secretion was quantified using an Enzyme-linked Lectin Assay (ELLA) in-house protocol detecting the carbohydrate groups of the collected mucus. 96-well plates were coated with 6 μg/mL Lectin from Triticum vulgaris (wheat) (Sigma, L0636) in PBS adjusted at pH 6.8 and incubated 1 h at 37°C. After washing steps with high 0.5M NaCl, 0.1% Tween-20 in PBS, samples, and standards (Mucin from porcine stomach Type II, Sigma, M2378) were incubated 30 min at 37°C. After washing, plates were incubated 30 min at 37°C with a detection solution containing 1μg/mL of Peroxidase conjugated Lectin from Glycine Max (soybean) (Sigma, L2650), in 0.1% BSA-PBS adjusted at pH 7.4. After the last washing steps, the TMB substrate reagent (3,3′,5,5′-tetramethylbenzidine purchased from ThermoFisher Scientific 34,021) was added and incubated for 10 min in the dark at RT. The reaction was stopped with 2N H_2_SO_4_ and the plates were read at 450 nm.

### 2.7. Muc5AC immunohistochemistry

Tissues were fixed using 4% formaldehyde in PBS with Ca^2+^/Mg^2+^ for 30 min. One epithelium per donor was evaluated for the presence of goblet cells with anti-Muc5AC-specific antibody (Abcam ab3649) and percentage of positive area were assessed. Briefly, inserts were processed for immunohistochemistry using four central transversal paraffin sections of 4 μm. Immunostaining of the slides was performed with the Benchmark automated platform (Ventana-Roche) and the Autostainer Link 48 (Dako) with the detection kit Ultraview DAB (DAB chromogeny).

The sections were pre-treated using heat-mediated antigen retrieval with sodium citrate buffer, pH 6, for 20 min. The sections were then incubated with primary Ab for 1 h at room temperature. A biotinylated secondary Ab (Dako) was used to detect the primary, and visualized using an HRP conjugated ABC system. DAB was used as the chromogen to reveal Muc5AC immune reaction. The sections were then counterstained with hematoxylin and mounted with DPX.

Image analysis using ImagePro Plus software (version 6.2, Media Cybernetics) was conducted to quantify goblet cells on four sections per insert. The whole images of stained sections were scanned and total DAB labeled dark areas were measured using “count and measure object” function based on dark brown color selection. The results are expressed as percentage of Muc5AC stained area of the total surface area of the epithelial sections. Data from the four sections were averaged for one insert.

### 2.8. Statistics

Results were expressed as scatter plots with the median. Statistical significance was calculated using ordinary one-way ANOVA (no matching), two-way ANOVA (no matching), with multiple comparisons or unpaired *t*-tests, and Spearman’s analysis for correlation analyzes using GraphPad Prism 7.02 software.

## 3. Results

### 3.1. All tested viruses replicate more robustly in respiratory tissues originating from asthmatic compared to control patients

Viral stocks were prepared and titrated in HAE to avoid cell-adaptation. Viruses (MOI of ~0.001) were applied at the apical surface of the tissues and removed after 4 h by extensive washes. RNA was extracted from apically released viruses and quantified by RT-qPCR as previously described (Essaidi-Laziosi et al., 2018).

**Figure 1:**
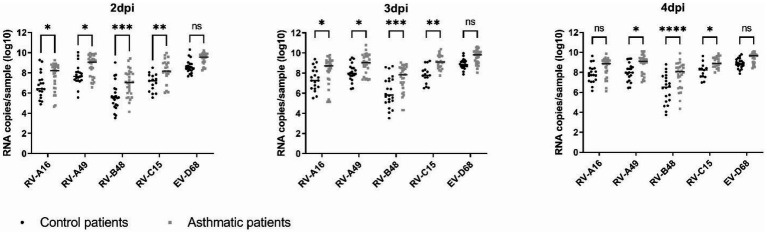
Virus production at the apical side of ALI culture of reconstituted HAE derived from non-asthmatic (control) or asthmatic patients represented as scatter plots with medians. Twelve control and 14 asthmatic donors were included (Supplementary Table S1). Statistically significant differences between HAE from asthmatic or control donors are indicated. ns: non-significant, *p < 0.05, **p < 0.01, ***p < 0.001, and ****p < 0.0001.

Replication was greater for EV-D68 and lower for RV-B48 compared to all other viruses independently of the condition (Figure 1), as previously shown (Essaidi-Laziosi et al., 2018). Interestingly, for all viruses, viral replication was increased in tissues from asthmatic patients compared to controls. This difference was observed independent on the tissue origin [nasal or bronchial (Supplementary Table S1)]. A C529Y mutation in *CDHR3* has been shown to increase asthma susceptibility. We sequenced this allele and found 5/12 (42%) and 6/14 (43%) donors, from the control and asthma groups, respectively, heterozygous for the asthma susceptibility allele and one control donor homozygous. No association between the presence of the susceptibility allele and viral growth could be observed (Supplementary Figure S1).

### 3.2. RV receptor expression does not account for the increased replication observed in tissues from asthmatic donors

**Figure 2:**
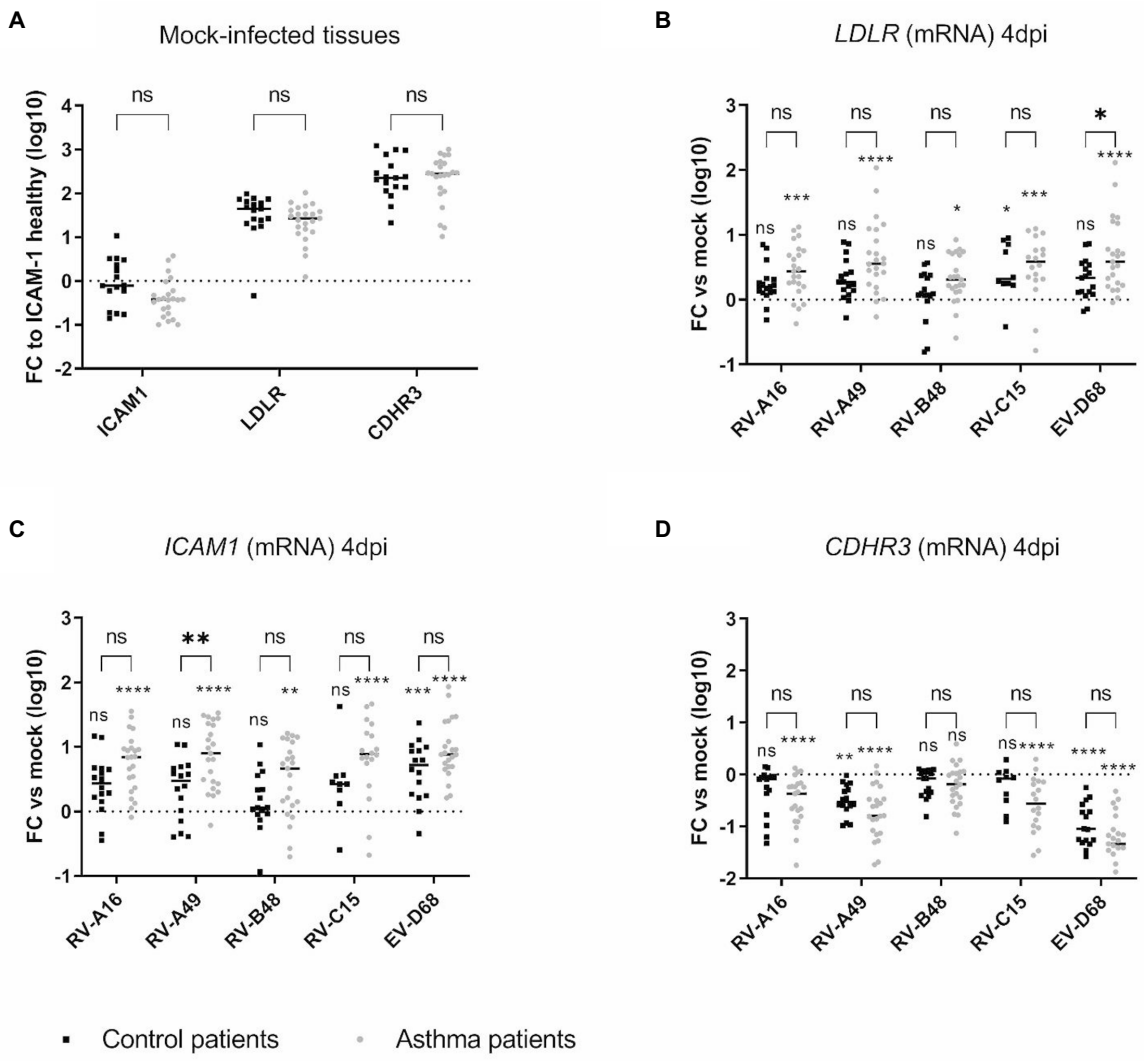
Expression of the different RV receptors in **(A)** mock-infected and in **(B–D)** infected tissues derived from asthmatic and control donors and measured by RT-sqPCR on total tissue lysates. In **(A)** the fold change (FC) is calculated relative to *ICAM1* expression in tissues derived from control donors. In **(B)**, **(C)**, and **(D)**, the fold change of *LDLR*, *ICAM1,* and *CDHR3* is calculated relative to expression in mock-infected tissues derived from donors within the same group (asthmatic or control). Eleven control and 12 asthmatic patients were included (Supplementary Table S1). The signs directly above each scatter plot indicate significance between mock-infected and infected tissues for each of the condition (asthma or control). The enlarged signs indicate significant differences between control and asthmatic donors. ns: non-significant, *p < 0.05, **p < 0.01, ***p < 0.001, and ****p < 0.0001.

We next assessed whether increased receptor expression could account for the enhanced replication observed in tissues from asthmatic donors. ICAM1, LDLR, and CDHR3 mRNA levels were quantified in non-infected or infected tissues derived from asthmatic or control donors by RT-sqPCR and no significant difference was observed between their basal levels (Figure 2A). This absence of difference in basal expression levels of RV receptors in tissues from control or asthmatic patients speaks against their involvement as an initial trigger of the increased viral replication observed in tissues from asthmatic donors. Of note, *ICAM1* basal expression is lower than *LDLR* and *CDHR3* (1 to 2 logs) and *CDHR3* expression is higher than *LDLR* (Figure 2A).

In contrast, in epithelia infected for 4 days (Figures 2B–D), *LDLR* and *ICAM1* expression was induced by the infection and this induction was significantly stronger in asthmatic donors. Correlation analysis (Supplementary Table S2) further highlighted a significant positive correlation between *LDLR* and *ICAM1* expression and the replication of RV-A49 and RV-B48 in tissues derived from healthy donors and between *LDLR* expression and replication of RV-C15 in tissues derived from asthmatic donors. Conversely, infection induced the downregulation of *CDHR3* and more significantly in asthmatic donors (Figure 2D). Accordingly, a significant negative correlation was found between *CDHR3* level and the replication of RV-A16, RV-A49, RV-B48, and RV-C15 in tissues derived from asthmatic donors (Supplementary Table S2). Of note, changes in receptor expression levels induced by RV-B48 were smaller in both control tissues and tissues from asthmatic donors. These observations were confirmed at the protein level by western blot (Supplementary Figure S2). Again, these data and the correlation analyzes do not support a causative role of receptor expression levels in the observed increased replication in tissues derived from asthmatic donors. This is particularly relevant for CDHR3, for which a decreased expression in tissues from asthmatic donors correlated with increased RV-C15 replication. As variation in receptor expression levels follows replication levels, it seems a consequence rather than a cause of the high replication observed in tissues from asthmatic donors.

### 3.3. Antiviral response is more important in infected tissues from asthmatic donors

**Figure 3:**
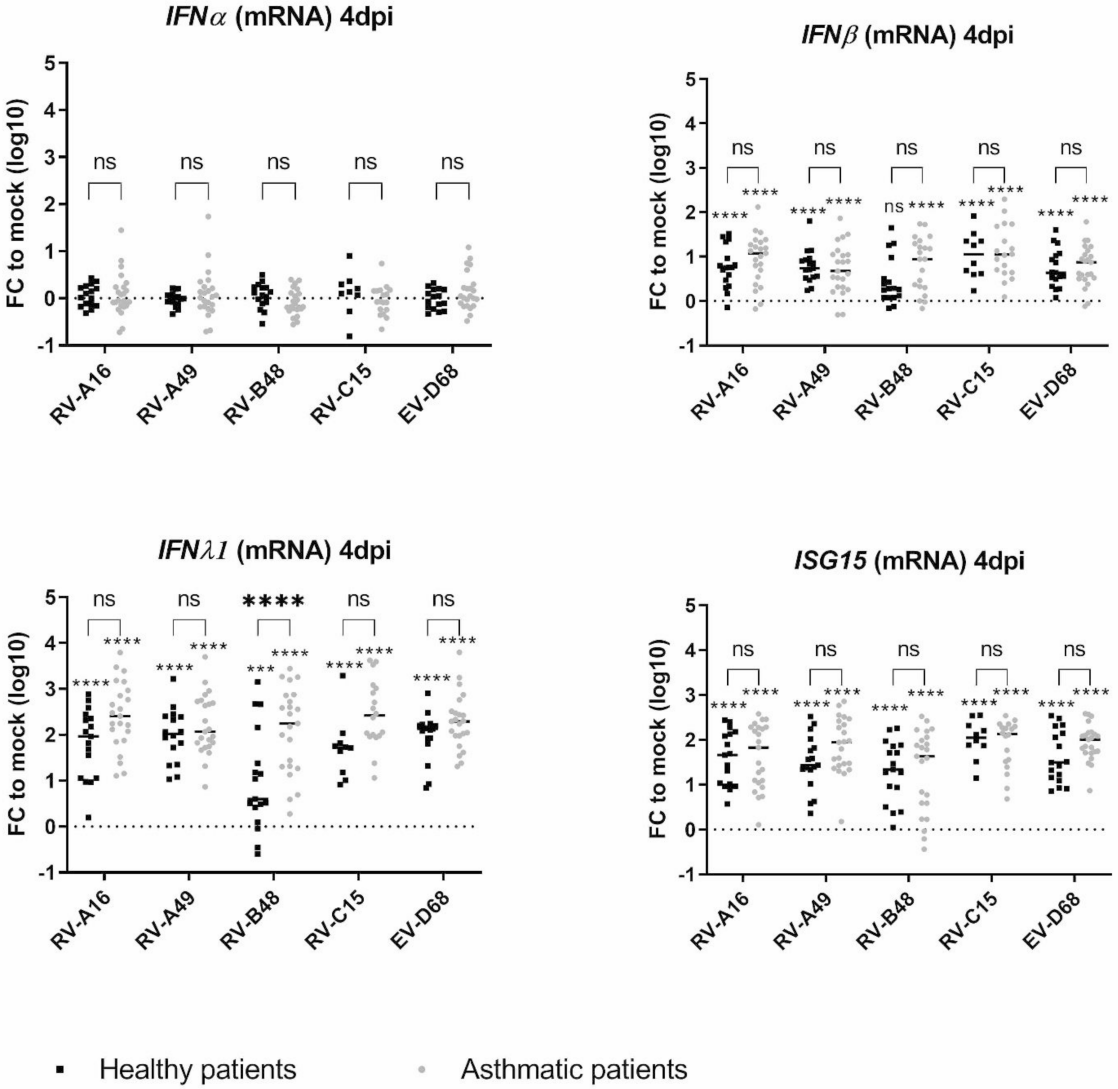
Expression of type I IFN, type III IFN, and ISG15 in infected tissues derived from asthmatic and non-asthmatic donors and measured by RT-sqPCR on total tissue lysates. The fold change (FC) is calculated relative to expression in mock-infected tissues derived from donors within the same group (asthmatic or control). Eleven control and 12 asthmatic patients were included (Supplementary Table S1). The signs directly above each scatter plot indicate significance between mock-infected and infected tissues for each of the condition (asthma or control). The enlarged signs indicate significant differences between control and asthmatic donors. ns: non-significant, *p < 0.05, ***p < 0.001, and ****p < 0.0001.

Type I and type III IFN-responses and induction of the interferon-stimulated gene 15 (*ISG15*) were compared by RT-sqPCR in control and tissues from asthmatic donors, 4 dpi (Figure 3). While *IFNα* was almost not induced in both tissue types, in line with the low induction of this cytokine in infected respiratory tissues (Essaidi-Laziosi et al., 2018; Filipe et al., 2022), *IFNβ*, *IFNλ1* and *ISG15* were significantly induced with a significantly higher *IFNλ1* induction in tissues from asthmatic donors. This increase was confirmed in a subset of tissues (Supplementary Table S1) by IFNλ1/λ3-cytokine measurement in the basal medium collected from day 1 to 4 pi (Supplementary Figure S3). The increased antiviral effector production was striking for RV-B, probably due to the significantly higher viral production in tissues from asthmatic donors (Figure 1). Correlation analysis indeed highlighted a significant positive correlation between RV-A49 viral load and IFNλ induction in tissues from healthy donors and between RV-B48 viral load and both IFNλ and β induction in tissues from both healthy and asthmatic donors (Supplementary Table S2). In conclusion, we observed higher rather than lower IFN-induction in tissues from asthmatic donors and the level of induction seems to follow viral replication trend.

### 3.4. Transcriptomic analysis highlights structural differences in the response to infection in tissues from asthmatic or control patients

**Figure 4:**
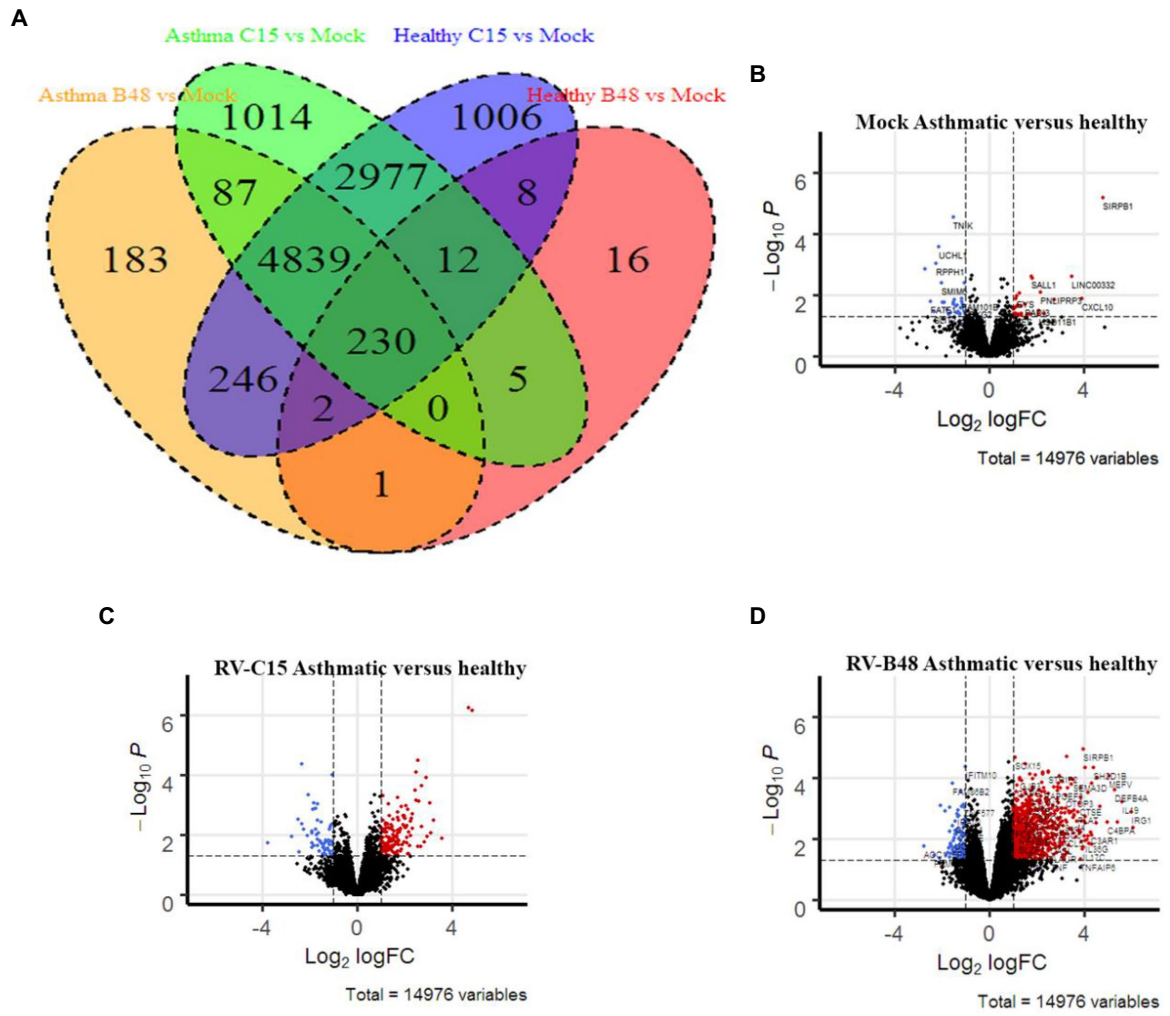
Differential gene expression in infected and mock-infected tissues derived from control versus asthmatic donors. **(A)** Venn diagram comparing the significant gene induction, relative to mock, in RV-B48 and RV-C15-infected tissues from control versus asthmatic donors. **(B–D)** Volcano plots summarizing the differential gene expression between control and asthmatic donors in **(B)** mock-infected, **(C)** RV-B48-infected, and **(D)** RV-C15-infected tissues. Red and blue dots correspond, respectively, to significantly upregulated and downregulated genes (*p*-value < 0.05). Dotted vertical line indicates fold differences of 2, dotted horizontal lines indicate significance at a nominal *p*-value of 0.05.

We performed a comparative transcriptomic analysis of tissues from control or asthmatic patients in the presence or absence of viral infection. We previously reported modifications of tissue metabolism and activation of innate immunity by RV-C15 and RV-B48 in control tissues (Essaidi-Laziosi et al., 2018). To compare the epithelial response of tissues derived from asthmatic donors, a larger transcriptomic analysis was carried out at 3dpi with the same viruses in tissues from control or asthmatic donors. RV-C15 induced more genes than RV-B48 in control tissues (9,320 versus 274 genes) (Figure 4A) as previously reported (Essaidi-Laziosi et al., 2018). Gene-induction by RV-B48 drastically increased in tissues from asthmatic donors (5,588 versus 271 genes), reflecting changes in replication levels. Differential gene expression was considerably higher in infected tissues compared to mock-infected tissues (Figures 4B–D).

**Figure 5:**
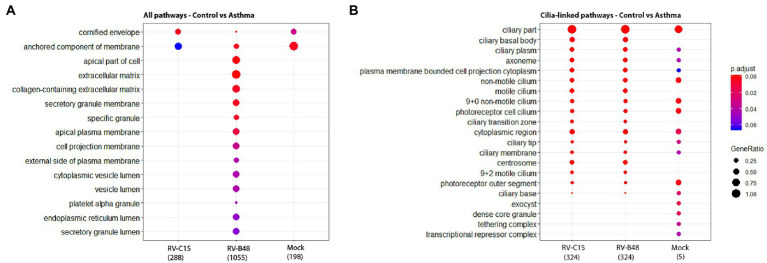
Enrichment of cellular pathways in infected and mock-infected tissues from control versus asthmatic donors. The top 15 of the most significantly enriched gene ontology cellular components analyzed from the total reads **(A)** and from reads of genes involved in the differentiation and function of ciliated cells **(B)** are shown, comparing epithelia originating from control versus asthmatic donors (Fold change >1, *p* < 0.05) in mock, RV-B48 and RV-C15 infections.

Pathway enrichment analysis in tissues from controls versus asthmatic donors using Gene Ontology highlighted modified pathways. In non-infected tissues, enriched components were mostly related to envelope and membrane composition (Figure 5A). More focused comparison of genes involved in the differentiation and function of ciliated cells highlighted striking differences between asthmatic and control donors (Figure 5B). Similar pathways were differentially enriched after infection, particularly for RV-B. Again, the higher enrichment for RV-B is in line with the enhanced replication observed in asthmatic donors for this virus.

### 3.5. Tissues from asthmatic patients exhibit reduced MCC and increased mucus secretion

**Figure 6:**
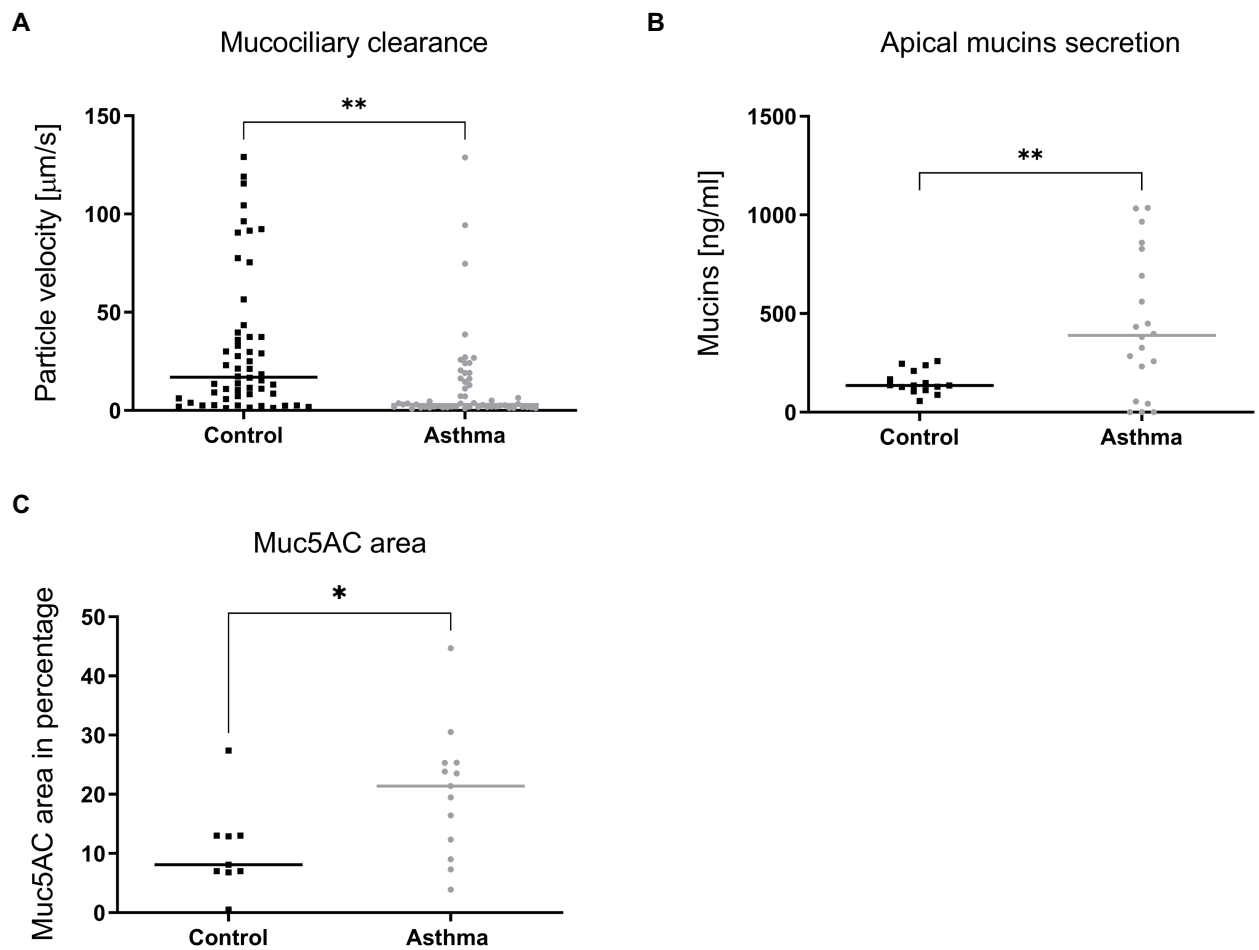
Mucociliary clearance **(A)**, mucus secretion **(B)** and expression of Muc5AC in immunostained tissue sections **(C)** compared in tissues derived from control or asthmatic donors. In **A**, 9 controls (54 inserts) and 7 asthmatic (55 inserts) donors were included in the comparison. In **B**, mucins were quantified from apical samples collected after 24h from tissues derived from 4 controls (15 inserts) and 5 asthmatic (20 inserts) donors. In **C**, tissues derived from 9 control and 9 asthmatic donors were processed. Statistically significant differences between HAE from asthmatic or control donors are indicated. *p < 0.05; **p < 0.01.

As most changes between asthmatic and controls (infected or not) were linked to tissue structure rather than induced tissue response, we compared the histology of tissues derived from healthy or asthmatic donors (Supplementary Figure S4A), as well as the tubulin expression and subcellular localization in infected or uninfected tissues (Supplementary Figure S4B). We did not observe macroscopic differences between the two groups. Since changes in transcriptomic profiles were already present in non-infected tissues (Figure 4; Essaidi-Laziosi et al., 2018), we then compared the MCC and mucus secretion in multiple non-infected tissues originating from a panel of distinct donors (Supplementary Table S1). As shown in Figure 6, tissues derived from asthmatic donors display a significantly decreased MCC and a significantly increased mucus secretion, confirmed by immunostaining of the muc5AC protein.

## 4. Discussion

In this study, we compared infections by RV-A major and minor group, RV-B, RV-C, and EV-D68, in HAE derived from asthmatic or control patients. First, we observed that RVs of all 3 species and, to some extent EV-D68, show increased replication in tissues from asthmatic patients and this increase is inversely associated with the replication of each virus in healthy tissues, with EV-D68 and RV-B48 showing the lowest and highest increase, respectively. The enhanced replication of RV-B48 in tissues from asthmatic donors was unexpected as this virus is less frequently associated with asthma exacerbation (Choi et al., 2021). However, RV-B replication and IFN-induction remained below the levels of the other viruses. In addition, the lower number of RV-B types (32 RV-B versus 80 RV-A and 57 RV-C) may also account for its lower detection in asthma exacerbations.

This overall effect on replication, independent of the species analyzed, made a causal association with viral receptor usage unlikely. Indeed, we observed no difference in basal receptor expression in the two groups of tissues. Nevertheless, after infection, ICAM1 and LDLR, the major and minor group receptors, were significantly induced, and this induction was proportional to viral replication levels. ICAM1 and LDLR are induced by inflammation (Bianco et al., 2000; Zhang et al., 2016) explaining these results. However, correlation analyzes did not highlight a positive correlation between RV-A16, RV-B48, and RV-A49 viral loads and expression of their respective receptor ICAM1 or LDLR, in tissues derived from asthmatic donors. In contrast, CDHR3 expression was decreased and the decrease was inversely proportional to the replication level, with a significant negative correlation observed for all viruses (except EV-D68) in tissues derived from asthmatic donors, even for RV-C15 whose replication is increased in this group. Because CDHR3 is expressed on ciliated cells, the primary target of RVs and EV-D68, this decrease is likely related to disruption of ciliated cell metabolism and/or ciliated cell death. Overall, these data indicate that different baseline expression levels of receptors do not represent the initial trigger for increased replication in tissues derived from asthmatic donors. However, after the onset of infection, the higher expression of ICAM1 and LDLR could possibly favor multiplication of major and minor group RVs in tissues from asthmatic donors. The situation differs for RV-Cs, where decreased CDHR3 expression would instead limit viral spread.

Concerning CDHR3, we also assessed the impact of the rs6967330 SNP on viral replication. We identified, respectively, 5/12 and 6/14 tissues from control and asthmatic donors heterozygous for the asthma susceptibility allele plus one homozygous control. We did not observe any significant increase in RV-C replication in presence of the *CDHR3* susceptibility allele. Our data thus contradict previous studies performed in similar models, where increased RV-C binding and/or replication was observed (Basnet et al., 2019; Everman et al., 2019). This may be due to the differentiation stage of the tissues at the time of infection. Basnet and colleagues have shown that the difference of expression between the mutated and non-mutated allele is higher before 21 days of tissue differentiation (Basnet et al., 2019). Everman and colleagues (Everman et al., 2019) also highlighted a greater CDHR3 expression in differentiating airway epithelial cells. Both studies showed increased RV-C replication using in-house developed tissues less differentiated than the commercially available, fully differentiated tissues used in this study. Additional investigations are needed to assess the true impact of the *CHDR3* mutation on RV-C replication in fully differentiated HAE from asthmatic donors. Apart from its role as RV-C receptor, CDHR3 is involved in the differentiation of ciliated airway cells (Lutter and Ravanetti, 2019) and participates in the tissue barrier function (Basnet et al., 2019; Everman et al., 2019). Its involvement in asthma exacerbation may thus rely on several pleiotropic effects.

We next assessed whether a deficient IFN-response could account for the increased viral replication. As in our previous study conducted on HAE of control donors (Essaidi-Laziosi et al., 2018), we observed differential replication and IFN-induction between the studied strains, with RV-B48 inducing a weak IFN-response compared to the other viruses. We also confirmed that compared to IFNλ, IFNβ is weakly induced and IFNα almost not expressed from infected HAE. An observation confirmed by others using primary bronchial epithelial cells (Liew et al., 2022). We could not highlight a reduced IFN-response in tissues from asthmatic donors, in contrast, this induction turned to be higher at 4 dpi due to the increased viral replication. Our data on increased RV replication in asthmatic conditions are in line with many studies (Xatzipsalti et al., 2008; Jackson et al., 2014; Hansel et al., 2017; Dhariwal et al., 2021), while others do not observe increased replication (Kennedy et al., 2014; Heymann et al., 2020). Data on IFN-response remain also debated. It is widely accepted that asthma exacerbation after RV infection is in part related to a deficient IFN-response which, in turn, leads to T2-inflammation (Duerr et al., 2016). Nevertheless, many studies, particularly in adult populations, did not observe such a deficient IFN-response, or even found an increased response (Krammer et al., 2021; Yang et al., 2021; Jackson and Gern, 2022; Liew et al., 2022). These inconsistent observations may rely on the baseline level of asthma control and severity between studies, the population age, the model used, and/or the experimental settings. We are using fully differentiated commercially available HAE derived from nasal or bronchial biopsies of adult patients. Furthermore, this model lacks immune cells and as such, the complex interplay between infected epithelial cells and neighbor immune cells. It is thus not fully adapted to study innate response in its entirety. In addition, we measured IFN mRNA at 4 dpi and IL28/29 cytokine from 1 dpi. Some studies report a delayed IFN-response rather than a decreased one (Veerati et al., 2020). We cannot exclude that in our setting, antiviral response was delayed before 24 hpi, allowing increased replication which in turn resulted in higher IFN-induction. This hypothesis is supported by a recent experimental infection with RV-A16 highlighting strong IFN-response at 4 dpi in asthmatic patients (Farne et al., 2022). Nevertheless, if defective very shortly after infection, this IFN-response was very strong afterward and even stronger in asthma patients. At later time points, IFN-induction appears thus to be a consequence rather than a cause of the differences in viral replication between asthma and controls.

As neither receptor expression nor IFN-induction after 24 hpi could consistently account for the overall increased viral replication observed in tissues from asthmatic patients, we performed unbiased transcriptomic analysis of tissues, infected or not with RV-B48 and RV-C15. This analysis highlighted basic morphologic and physiologic differences between tissues derived from controls or asthmatic donors, even in absence of infection. These data were supported by diminished MCC in asthma-derived tissues. The critical role of MCC to limit viral infections is well established and patients presenting defective MCC (due to genetic or environmental causes) suffer from repeated infections (Adivitiya Kaushik et al., 2021). Altogether our observations suggest that the intrinsic defense mechanisms of the respiratory mucosae are affected in tissues derived from asthmatic patients, increasing permissiveness and susceptibility to RV or EV-D68 infections. Perturbation of the epithelial barrier function was observed previously in asthmatic patients (Xiao et al., 2011; Looi et al., 2018). Similarly, mucus hypersecretion was reported in asthmatic settings (Rogers and Barnes, 2006; Evans et al., 2009; Martinez-Rivera et al., 2018). Interestingly, this defective barrier function and perturbed mucus secretion are retained in this *ex vivo* reconstituted tissue culture model, derived after over 45 days from isolated cells taken out of the context of the asthmatic host. This strongly indicates an involvement of genomic imprinting mechanisms, as suggested in the scientific literature (Gruzieva et al., 2021).

To conclude, we could show increased replication of RV-A major and minor groups, RV-B, RV-C, and EV-D68 in HAE derived from asthmatic patients. Our data highlight that a global disruption of epithelial cell barrier function, rather than changes in receptor expression or a deficient IFN response, is likely a key factor in the increased permissiveness and susceptibility to RV or EV-D68 infections. All in all, this work provides novel insights into the mechanism underlying asthma exacerbations upon respiratory enterovirus infections.

## Data availability statement

The data presented in the study are deposited in the NCBI website with the GEO accession number: GSE222129. Access: https://www.ncbi.nlm.nih.gov/geo/query/acc.cgi?acc=GSE222129.

## Ethics statement

The studies involving human participants were reviewed and approved by commission cantonale d’éthique de la recherche CCER from Geneva. The patients/participants provided their written informed consent to participate in this study.

## Author contributions

ME-L, LR, and CT contributed to conception and design of the study. ME-L, LR, BB, IP, and SoC performed the experimental work. ME-L, FP-R, and NH ran the bioinformatic part. LK, SH, and SaC contributed to tissue collection and/or development. ME-L and LR wrote the first draft of the manuscript. CT revised the manuscript. All authors contributed to the manuscript revision, and read and approved the submitted version.

## Funding

The study was supported by the Swiss National Foundation (SNF) (Grant 310030_184777 to CT), the SNF Marie Heim-Vögtlin subsides (Grant PMPDP3-158269 and PMPDP3-158269 to ME-L), and the OrganoVIR (grant 812673) in the European Union’s Horizon 2020 programme, and the Sandoz foundation (LR Salary).

## Conflict of interest

BB, SH and SaC are employed by Epithelix company.

The remaining authors declare that the research was conducted in the absence of any commercial or financial relationships that could be construed as a potential conflict of interest.

## Publisher’s note

All claims expressed in this article are solely those of the authors and do not necessarily represent those of their affiliated organizations, or those of the publisher, the editors and the reviewers. Any product that may be evaluated in this article, or claim that may be made by its manufacturer, is not guaranteed or endorsed by the publisher.

## Footnotes

^1^http://www.epithelix.com/products/mucilair
