# Supplementary material for: Altered cell function and increased replication of rhinoviruses and EV-D68 in airway epithelia of asthma patients
Source: Front Microbiol. 2023 Mar 1;14:1106945. doi: 10.3389/fmicb.2023.1106945 (PMC10014885; doi:10.3389/fmicb.2023.1106945)
Supplement: Supplementary file 1 [file Data_Sheet_1.docx]

Supplementary Material

Altered cell function and increased replication of rhinoviruses and EV-D68 in airway epithelia of asthma patients

Manel Essaidi-Laziosi^1^, Léna Royston^1^, Bernadett Boda^2^, Francisco Javier Pérez-Rodriguez^1,3^, Isabelle Piuz^1^, Nicolas Hulo^4^, Laurent Kaiser^3^, Sophie Clément^1^, Song Huang^2^, Samuel Constant^2^, Caroline Tapparel^1*^

^1^Department of Microbiology and Molecular Medicine, Faculty of Medicine. University of Geneva, Geneva, Switzerland

^2^Epithelix Sàrl, Plan les Ouates, Geneva, Switzerland

^3^Division of Infectious Diseases, Geneva University Hospital, Geneva, Switzerland

^4^Service for Biomathematical and Biostatistical Analyses. Institute of Genetics and Genomics, University of Geneva, Geneva, Switzerland

*** Correspondence:**Corresponding Author: caroline.tapparel@unige.ch

# Supplementary Materials and Methods

## Histology paraffin-embedded tissue sections

Paraffin was removed from tissue sections by embedding the sections twice in UltraClear solution (3905.5000PE, Biosystems, Switzerland) for 3 min and twice in EtOH 96% (E/0600DF/15, Fisher Chemical, Thermo Fisher Scientific, Switzerland) for 3 min. Tissue sections were counterstained with Mayer’s Hematoxylin Solution (5 min), eosin (7 min) and mounted with Neo Mount medium (1.09016.0500, Merck, Switzerland). Images were acquired with Olympus VS120 microscope using a U Plan S Apo 100x/1.4 Oil objective.

## Immunofluorescence

Infected tissues and controls were washed 3 times with PBS and fixed for 30’ in 4% paraformaldehyde at room temperature (RT). Tissues were washed 3 times with PBS, permeabilized with Perm/wash buffer (BD 554723), and washed with PBS before incubation with rabbit anti-beta IV tubulin Ab (Abcam179504, diluted 1/250). After intensive washing with PBS, Alexa 488-goat anti-rabbit Ab (Life technologies A11008, diluted 1/3000) was added for 45’ at 37°C. After rinsing with PBS, tissues were stained with 4', 6-diamino-2-phenylindole (DAPI), washed with PBS and mounted onto glass slides in Fluoroprep (BioMerieux). Images were acquired with a Zeiss LSM 700 Meta confocal microscope with a 63.6/1.4 objective, processed by Imaris and are presented in 3D projections.

# Supplementary Figures and Tables

## Supplementary Figures


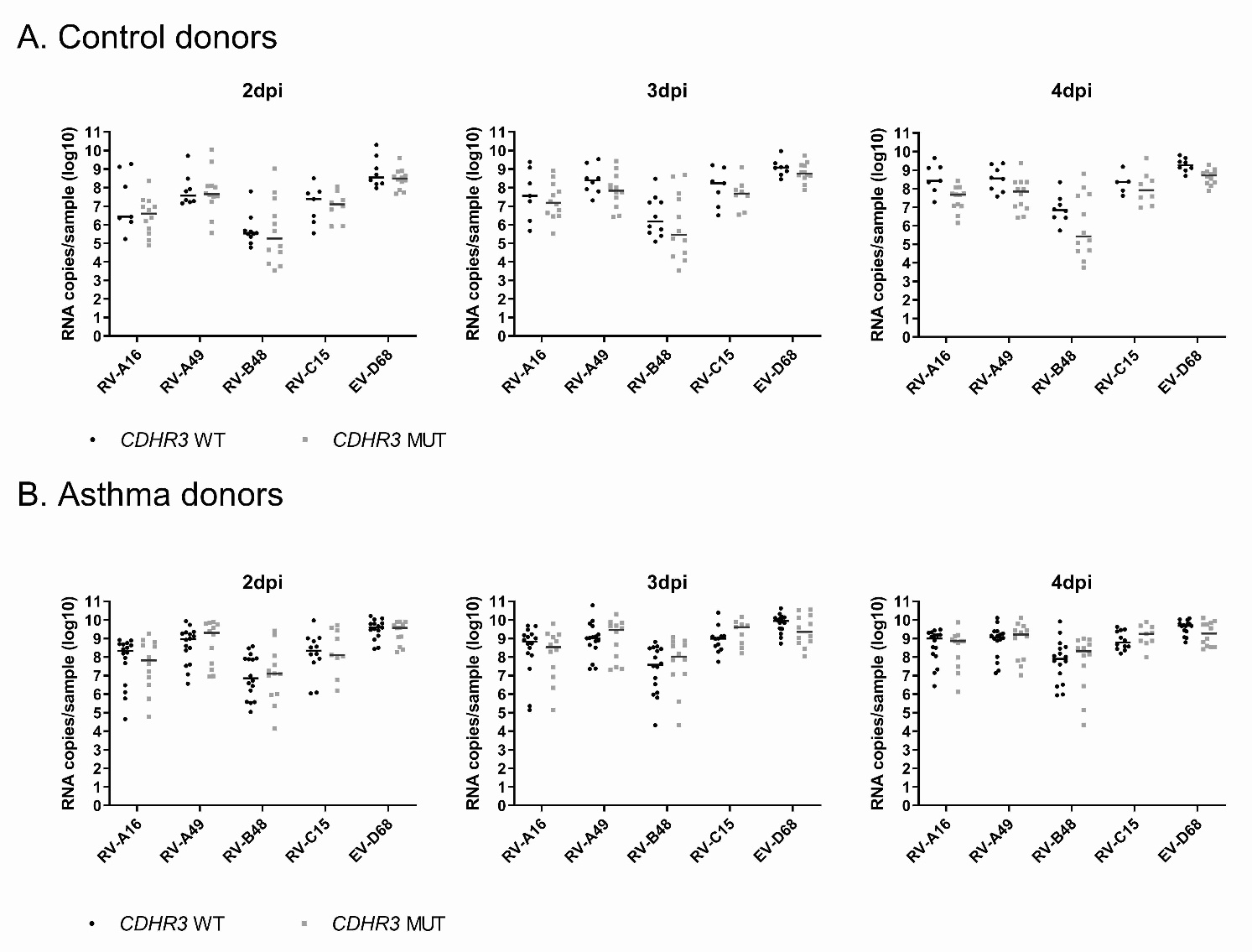


**Supplementary Figure 1.** Figure S1. Virus production at the apical side of ALI cultures of reconstituted HAE derived from control (A) or asthmatic patients (B) grouped based on their CDHR3 allele and represented by as scatter plots with the median. In A, n=5 donors with wt and 7 donors with mutated CDHR3. In B, n= 8 donors with wt and 6 donors with mutated CDHR3 (Table S1). No statistically significant differences were observed between groups.

**
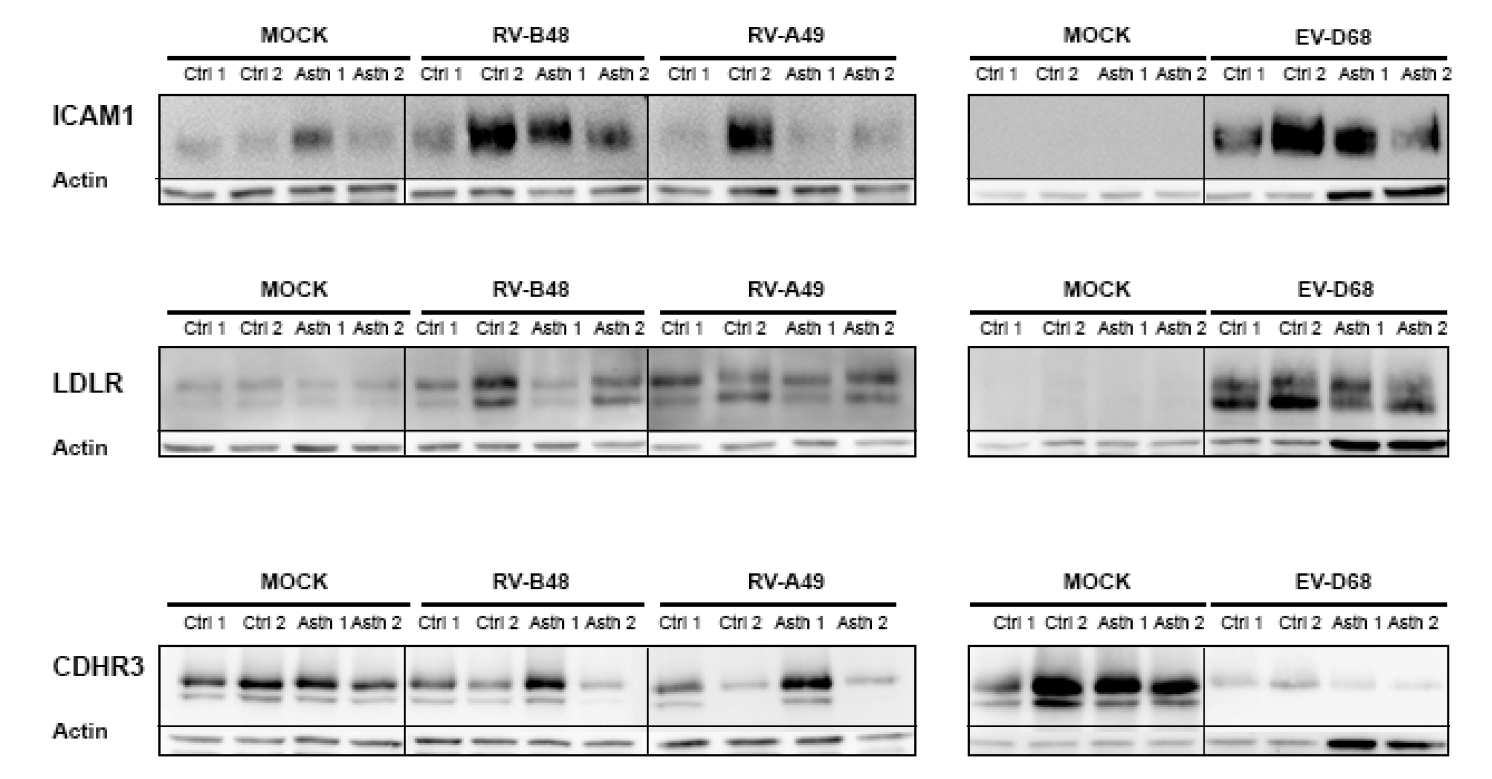
**

**Supplementary Figure 2.** Figure S2. Expression of the different RV receptors assessed by western blot in tissues originating from two control (C1 and C2) or two asthmatic (A1 and A2) donors (Table S1) and either non-infected (Mock) or infected for four days with RV-B48 or RV-A49. Beta-actin was used as internal control.

**
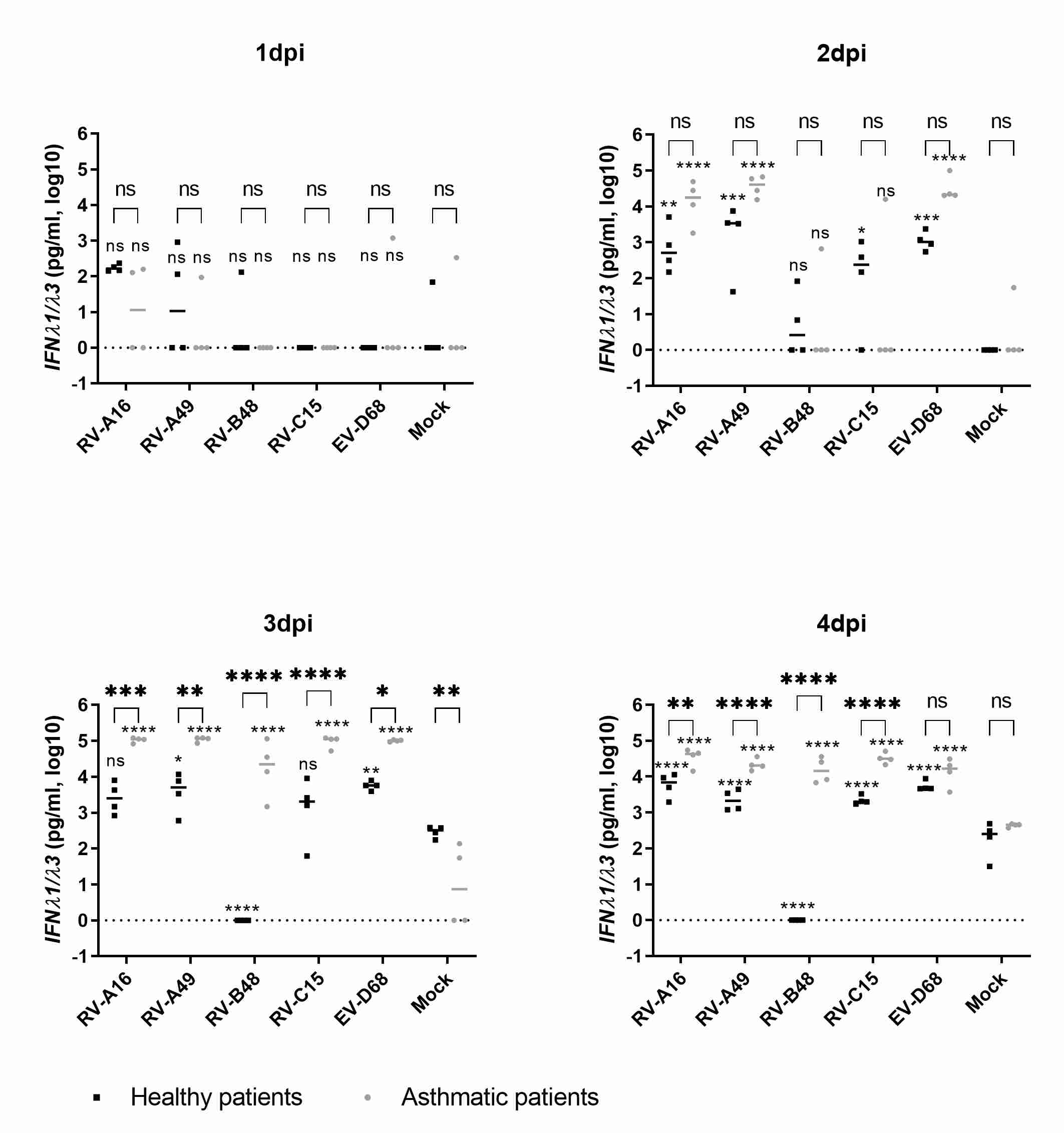
**

**Supplementary Figure 3.** Figure S3. IFNλ1/3 protein concentration (pg/ml) measured by ELISA in the basal medium at 1, 2, 3 and 4dpi with the indicated virus. 4 control and 4 asthmatic patients were included (Table S1). Data are represented as scatter plots with medians. Statistical significance was calculated using the two-way ANOVA. The signs directly above each scatter plot indicates significance relative to mock-infected tissues with the same condition (asthma or control). The enlarged signs indicate significant differences between control and asthmatic donors. ns: non-significant, ****P<0.0001, **P< 0.01, *P< 0.05.

**
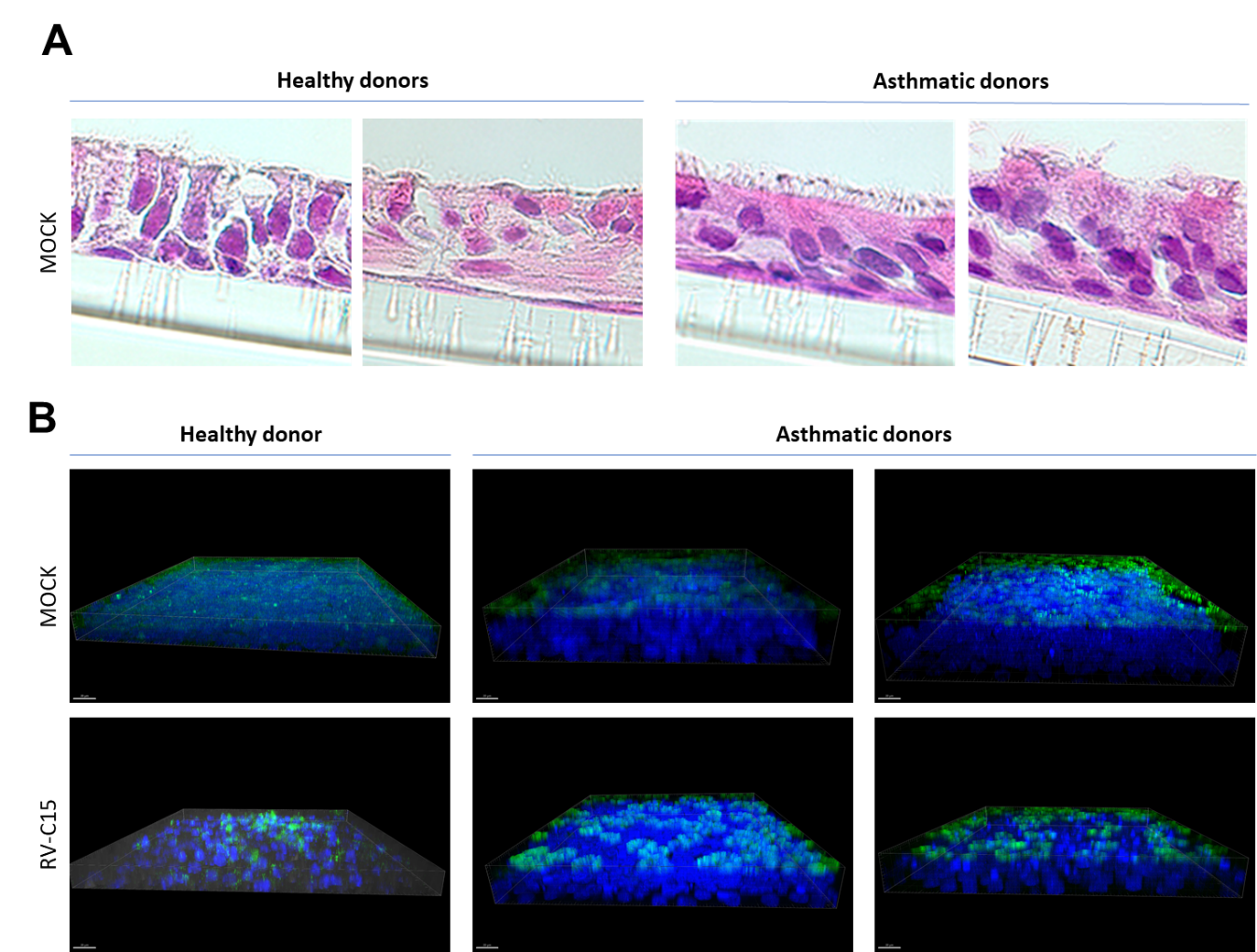
 Supplementary Figure 4.** Figure S4. (A) Sections of non-infected (MOCK) tissues derived from asthmatic or healthy donors stained with hematoxylin and Eosin. (B) Immunofluorescence of epithelia derived from healthy or asthmatic donors and infected with RV-C15 or non-infected. Three-dimensional view of non-infected and infected tissues, showing ciliated cells stained with anti-tubulin antibodies (green), and cell nuclei in blue. Immunofluorescence was performed at day 4 after infection.

## Supplementary Tables

| **Condition** | **Donor** | **Tissus origin** | **Gender** | **Age** | **CDHR3 AA_529_** | **Disease** | **Treatment** | **Assay** | | | | | |
| --- | --- | --- | --- | --- | --- | --- | --- | --- | --- | --- | --- | --- | --- |
|  |  |  |  |  |  |  |  | **Replication** | **Receptor mRNA /Western** | **Innate response mRNA/Elisa** | **MCC** | **Mucins** | **Muc 5AC** |
| **HEALTHY** | pool7 (14 donors) | Nasal |  |  | C/C | no pathlology | none | x | x | x |  |  |  |
|  | MD059701 | Nasal | M | 24 | C/C | no pathlology | none | x | x | x/x |  |  |  |
|  | MD005501 | Nasal | M | 42 | C/Y | no pathlology | none | x | x | x/x |  |  |  |
|  | MD040201 | Nasal | M | 71 | C/Y | no pathlology | none | x | x | x/x |  |  |  |
|  | MD047101 | Nasal | F | 40 | Y/Y | no pathlology | none | x | x | x/x |  |  |  |
|  | *AB059101 | Nasal | M | 76 | C/C | no pathlology | none | x |  |  |  |  |  |
|  | *AB063501 | Nasal | M | 36 | C/C | no pathlology | none | x | x | x |  |  |  |
|  | *AB021901 | Nasal | M | 66 | C/C | no pathlology | none | x | x | x |  |  |  |
|  | MD0802 | Bronchial | F | 55 | C/Y | no pathlology | none | x | x | x |  |  |  |
|  | MD0436 | Nasal | M | 46 | C/C | no pathlology | none | x | x/x | x |  |  |  |
|  | MD0774 | Nasal | M | 38 | C/Y | no pathlology | none | x | x/x | x |  |  |  |
|  | MD0782 | Bronchial | F | 61 | C/Y | no pathlology | none | x | x | x | x | x |  |
|  | MD077501 | Bronchial | M | 19 | ND | no pathlology | none |  |  |  | x |  |  |
|  | MD080101 | Bronchial | M | 27 | ND | no pathlology | none |  |  |  | x |  |  |
|  | MD078701 | Bronchial | F | 56 | ND | no pathlology | none |  |  |  | x | x |  |
|  | MD079301 | Bronchial | M | 62 | ND | no pathlology | none |  |  |  | x | x |  |
|  | MD069301 | Bronchial | M | 65 | ND | no pathlology | none |  |  |  | x | x | x |
|  | MD076801 | Bronchial | F | 59 | ND | no pathlology | none |  |  |  | x |  | x |
|  | MD077201 | Bronchial | M | 63 | ND | no pathlology | none |  |  |  | x |  | x |
|  | MD068001 | Bronchial | F | 71 | ND | no pathlology | none |  |  |  |  |  | x |
|  | MD0670 | Bronchial | M | 15 | ND | no pathlology | none |  |  |  |  |  | x |
|  | MD048401 | Nasal | M | 55 | ND | no pathlology | none |  |  |  |  |  | x |
|  | MD051501 | Nasal | F | 46 | ND | no pathlology | none |  |  |  |  |  | x |
|  | MD069201 | Nasal | F | 58 | ND | no pathlology | none |  |  |  |  |  | x |
|  | MD072201 | Nasal | M | 61 | ND | no pathlology | none |  |  |  |  |  | x |
| **ASTHMA** | AB066501 | Bronchial | F | 55 | C/C | Asthma and Renal Carcinoma | ND | x |  |  |  |  |  |
|  | AB067701 | Bronchial | F | 50 | C/C | Asthma, Chronic | Albuterol, predisone | x |  |  |  |  |  |
|  | AB065801 | Nasal | M | 53 | C/Y | Asthma | Topical cortico | x | x | x |  |  |  |
|  | MD0792 | Bronchial | F | 77 | C/C | Asthma | Albuterol, ASA, B12 | x | x | x |  |  |  |
|  | AB050901 | Nasal | M | 57 | C/C | Asthma, allergic rhinitis | Systemic and topical cortico | x | x | x |  |  |  |
|  | *AB042101 | Nasal | M | 45 | C/C | Asthma, Sinusitis | Topical cortico | x | x | x |  |  |  |
|  | *AB050101 | Nasal | M | 44 | C/C | Asthma sinusitis allergic rhinitis | Topical cortico treatments | x | x | x/x |  |  | x |
|  | *AB041401 | Nasal | M | 33 | C/C | Asthma | Topical cortico | x | x | x/x |  |  | x |
|  | AB021402 | Nasal | M | 50 | C/Y | Asthma | Topical cortico | x | x | x/x |  |  | x |
|  | MD006501 | Bronchial | F | 48 | C/Y | Polyposis sinusitis, Asthma | Topical cortico, beclojet, foradil | x | x | x/x |  |  | x |
|  | MD048901 | Bronchial | F | 55 | C/Y | Asthma | ND | x | x | x | x |  | x |
|  | MD078901 | Bronchial | F | 17 | C/C | Asthma | ProAir inhaler, Dulera, Singulair | x | x | x | x | x |  |
|  | MD0809 | Nasal | M | 24 | C/Y | Asthma | ProAir inhaler, Dulera, Singulair | x | x/x | x |  |  |  |
|  | MD0807 | Nasal | M | 61 | C/Y | Asthma | ProAir inhaler, Dulera, Singulair | x | x/x | x |  |  |  |
|  | MD075801 | Bronchial | M | 40 | ND | Asthma since childhood | Unspecified inhaler, antibiotic for bacterial sinusitis |  |  |  | x |  |  |
|  | MD074001 | Bronchial | F | 67 | ND | Asthma since last year | Respiratory therapy |  |  |  | x | x | x |
|  | MD074402 | Bronchial | F | 47 | ND | Asthma | Inhaler |  |  |  | x | x | x |
|  | MD071601 | Bronchial | F | 41 | ND | Asthma (adult) | Singulair and steroid use for 10yrs |  |  |  | x |  | x |
|  | MD080001 | Bronchial | F | 54 | ND | Asthma | ND |  |  |  | x |  |  |
|  | MD075802 | Bronchial | M | 40 | ND | Asthma since childhood | Unspecified inhaler, antibiotic for bacterial sinusitis |  |  |  |  | x | x |

**Table S1:** Donor characteristics. *Tissues used for RNAseq. PPool: tissues developed from a pool of 14 different donors. Abbreviation: F, female. M, male. ND, not documented

|  |  | **IFN λ** | **IFNβ** | **ISG15** | **ICAM1** | **LDLR** | **CDHR3** |
| --- | --- | --- | --- | --- | --- | --- | --- |
| **RV-A16 Ctrl** | **Spearman R** | 0,406 | 0,335 | 0,329 | 0,068 | 0,453 | -0,215 |
|  | **P value** | 0,120 | 0,204 | 0,213 | 0,812 | 0,080 | 0,423 |
|  |  | ns | ns | ns | ns | ns | ns |
| **RV-A16 Asthma** | **Spearman R** | 0,298 | 0,174 | 0,281 | 0,401 | 0,132 | **-0,642** |
|  | **P value** | 0,167 | 0,426 | 0,194 | 0,058 | 0,548 | **0,001** |
|  |  | ns | ns | ns | ns | ns | ******* |
| **RV-A49 Ctrl** | **Spearman R** | **0,571** | 0,429 | 0,047 | **0,644** | 0,556 | -0,446 |
|  | **P value** | **0,023** | 0,087 | 0,861 | **0,009** | 0,022 | 0,074 |
|  |  | ***** | ns | ns | ****** | * | ns |
| **RV-A49 Asthma** | **Spearman R** | 0,238 | -0,040 | 0,087 | 0,207 | 0,244 | **-0,827** |
|  | **P value** | 0,275 | 0,858 | 0,693 | 0,344 | 0,262 | **<0,0001** |
|  |  | ns | ns | ns | ns | ns | ******** |
| **RV-B48 Ctrl** | **Spearman R** | **0,814** | **0,725** | 0,169 | 0,212 | **0,537** | -0,086 |
|  | **P value** | **0,0001** | **0,001** | 0,515 | 0,430 | **0,028** | 0,744 |
|  |  | ******* | ****** | ns | ns | ***** | ns |
| **RV-B48 Asthma** | **Spearman R** | **0,789** | **0,752** | 0,235 | 0,382 | 0,317 | **-0,549** |
|  | **P value** | **<0,0001** | **<0,0001** | 0,281 | 0,072 | 0,140 | **0,007** |
|  |  | ******** | ******** | ns | ns | ns | ****** |
| **RV-C15 Ctrl** | **Spearman R** | 0,346 | 0,370 | 0,576 | 0,067 | 0,018 | -0,346 |
|  | **P value** | 0,330 | 0,296 | 0,088 | 0,880 | 0,973 | 0,330 |
|  |  | ns | ns | ns | ns | ns | ns |
| **RV-C15 Asthma** | **Spearman R** | 0,152 | -0,261 | -0,450 | -0,386 | **-0,543** | **-0,743** |
|  | **P value** | 0,558 | 0,309 | 0,071 | 0,126 | **0,026** | **0,0009** |
|  |  | ns | ns | ns | ns | ***** | ******* |
| **EV-D68 Ctrl** | **Spearman R** | 0,360 | 0,179 | 0,319 | 0,047 | 0,414 | 0,110 |
|  | **P value** | 0,156 | 0,491 | 0,212 | 0,865 | 0,100 | 0,673 |
|  |  | ns | ns | ns | ns | ns | ns |
| **EV-D68 Asthma** | **Spearman R** | -0,070 | -0,007 | -0,026 | 0,084 | 0,303 | -0,279 |
|  | **P value** | 0,750 | 0,745 | 0,906 | 0,703 | 0,160 | 0,197 |
|  |  | ns | ns | ns | ns | ns | ns |

**Table S2:** Pearson’s correlation analyses of viral load, receptor and innate immunity modulators induction quantified by RT-qPCR in tissues derived from asthmatic (Asthma) or healthy (Ctrl) donors and infected for 4 days with the indicated virus. Statistical significance was determined with Spearman Analysis using GraphPad Prism 7 software (GraphPad Software, Inc., San Diego, CA, USA). ns: non-significant, *P< 0.05, **P< 0.01, ***P< 0.001, ****P< 0.0001.
